# Endocrine Disorders and COVID-19 Severity in Pediatric Populations: A Systematic Review

**DOI:** 10.7759/cureus.88458

**Published:** 2025-07-21

**Authors:** Afra Mohamed M Awad Abdu Alla, Hanady ME M Osman, Tayseer Ahmed Khalid Babikir, Alaa Hamed Hag Elzain Eltoum, Sitelgeel Ahmed Mohammed Alawad, Sally Hassan Ali Hassanin

**Affiliations:** 1 Pediatrics, Tuwaiq Medical Complex, Riyadh, SAU; 2 Quality and Patient Safety, Najran Armed Forces Hospital, Ministry of Defense Health Services, Najran, SAU; 3 Pediatrics, Newham University Hospital, London, GBR; 4 Pediatrics, Tipperary University Hospital, Tipperary, IRL; 5 Pediatrics, Maidstone and Tunbridge Wells NHS Trust, Royal Tunbridge Wells, GBR; 6 Pediatrics and Neonatal Intensive Care Unit, Al Ansari Specialist Hospital, Yanbu, SAU

**Keywords:** covid-19, diabetes mellitus, endocrine disorders, growth disorders, pandemic, pediatric endocrinology, sars-cov-2, systematic review, thyroid dysfunction

## Abstract

The COVID-19 pandemic has significantly impacted global health systems, with emerging evidence suggesting unique implications for pediatric populations with endocrine disorders. While children generally experience milder acute COVID-19 symptoms, those with pre-existing endocrine conditions may face heightened risks due to the interplay between viral infection and endocrine homeostasis. This systematic review aimed to synthesize evidence on the relationship between endocrine disorders and COVID-19 severity in children, focusing on disease outcomes, metabolic control, and management challenges during the pandemic. Following the Preferred Reporting Items for Systematic Reviews and Meta-Analyses (PRISMA) 2020 guidelines, a systematic search was conducted across PubMed, Scopus, Web of Science, and Cochrane Library. Ten studies meeting the inclusion criteria were selected after screening 638 records. Data were extracted on study characteristics, patient demographics, endocrine disorders, COVID-19 severity outcomes, and key findings. Risk of bias was assessed using the Newcastle-Ottawa Scale (NOS) tool. A narrative synthesis was performed due to heterogeneity in study designs and outcomes. The review revealed significant pandemic-related disruptions in pediatric endocrine health, including increased central precocious puberty cases and elevated BMI z-scores in children with obesity. Diabetes outcomes were mixed: type 1 diabetes patients had better mortality prognoses than type 2 diabetes patients, but diabetic ketoacidosis rates surged. Thyroid dysfunction and stable medication adherence in congenital adrenal hyperplasia were also noted. Risk of bias varied, with three studies rated low, five rated moderate, and two rated as high risk of bias. COVID-19 exacerbated endocrine disorders in children through direct viral effects and indirect lifestyle and healthcare disruptions. The findings underscore the need for adaptive care strategies, including telehealth and mental health support, to mitigate long-term impacts. Future research should prioritize prospective studies to evaluate sustained effects and interventions for at-risk populations.

## Introduction and background

The COVID-19 pandemic, caused by the novel coronavirus SARS-CoV-2, has presented unprecedented challenges to global healthcare systems, with emerging evidence suggesting unique implications for pediatric populations with endocrine disorders [1]. While children generally experience less severe acute COVID-19 manifestations compared to adults, those with pre-existing endocrine conditions may face compounded risks due to the intricate interplay between viral infection and endocrine homeostasis [2,3]. This systematic review examines the current evidence on how COVID-19 affects disease severity, metabolic control, and clinical management in children with endocrine disorders, addressing critical knowledge gaps in this vulnerable population.

Endocrine disorders in children, including diabetes mellitus, growth hormone deficiencies, thyroid dysfunction, and adrenal disorders, involve complex regulatory systems that may be disrupted by viral infections [4]. SARS-CoV-2 has demonstrated tropism for multiple endocrine tissues through angiotensin-converting enzyme 2 (ACE2) receptor expression [5], potentially exacerbating pre-existing conditions or unmasking latent endocrine dysfunction. The pandemic's secondary effects, including healthcare disruptions, lifestyle changes, and psychological stress, have further complicated disease management for pediatric endocrine patients [6,7]. Despite growing recognition of these challenges, comprehensive syntheses of evidence remain limited, particularly regarding differential impacts across various endocrine conditions and age groups.

This systematic review addresses critical questions about COVID-19's impact on pediatric endocrine disorders, including comparative disease severity across conditions, pathophysiological mechanisms linking SARS-CoV-2 to endocrine dysfunction, and the efficacy of alternative care models during healthcare disruptions. By synthesizing global evidence, we aim to inform clinical practice and public health strategies while identifying research gaps, with findings particularly relevant for clinicians and policymakers managing this vulnerable population. The review's implications extend beyond acute pandemic management, offering insights into viral-endocrine interactions and opportunities to transform chronic disease care paradigms, ultimately strengthening healthcare resilience for future public health crises.

## Review

Methodology

This systematic review was conducted in accordance with the Preferred Reporting Items for Systematic Reviews and Meta-Analyses (PRISMA) 2020 guidelines to ensure methodological rigor, transparency, and reproducibility [8].

Eligibility Criteria

The inclusion and exclusion criteria were established to identify studies that specifically examined the association between endocrine disorders and COVID-19 severity in pediatric populations. Table 1 outlines the detailed selection criteria.

**Table 1:** Eligibility criteria for studies selection.

Category	Inclusion criteria	Exclusion criteria
Population	Pediatric patients (ages 0–18 years) with pre-existing or newly diagnosed endocrine disorders	Adult populations (>18 years) or studies not stratifying pediatric data
Exposure	Confirmed or suspected COVID-19 infection	Studies not assessing COVID-19 or SARS-CoV-2 infection
Outcomes	COVID-19 severity (e.g., hospitalization, ICU admission, mortality, and metabolic complications)	Studies lacking primary outcome data related to endocrine dysfunction or COVID-19 severity
Study design	Observational studies (cohort, cross-sectional, case-control), randomized controlled trials, systematic reviews/meta-analyses	Case reports, non-peer-reviewed articles (editorials, conference abstracts), animal studies
Publication type	Peer-reviewed original research articles and systematic reviews	Preprints, commentaries, letters without original data
Language	No restrictions (non-English articles translated where feasible)	None
Time period	No date restrictions (all published literature up to search date considered)	None

Information Sources and Search Strategy

A systematic search was conducted across four major electronic databases: PubMed, Scopus, Web of Science, and Cochrane Library. The search strategy was developed in collaboration with a medical librarian to ensure sensitivity and specificity. The full search strategy for each database is given in Table 2. Additionally, manual searches of reference lists from included studies and relevant review articles were performed to identify additional eligible publications.

**Table 2:** Search strategy for four different databases.

Database	Search strategy
PubMed	(COVID-19 OR SARS-CoV-2 OR Coronavirus disease 2019) AND (Endocrine System Diseases[Mesh] OR endocrine disorder OR diabetes mellitus OR thyroid disease OR adrenal disorder OR pituitary disorder OR growth disorder OR metabolic syndrome OR obesity) AND (pediatric OR child OR adolescen OR infant OR youth OR teen) AND (severity OR clinical outcome OR hospitalization OR ICU OR intensive care OR mortality OR death OR complication)
Scopus	(TITLE-ABS-KEY ( "COVID-19" OR "SARS-CoV-2" OR "Coronavirus disease 2019" )) AND (TITLE-ABS-KEY ( "endocrine disorder" OR "diabetes mellitus" OR "thyroid disease" OR "adrenal disorder" OR "pituitary disorder" OR "growth disorder" OR "metabolic syndrome" OR "obesity" )) AND (TITLE-ABS-KEY ( pediatric OR child OR adolescen OR infant OR youth OR teen )) AND (TITLE-ABS-KEY ( severity OR "clinical outcome" OR hospitalization OR ICU OR "intensive care" OR mortality OR death OR complication ))
Web of Science	TS=(COVID-19 OR SARS-CoV-2 OR "Coronavirus disease 2019") AND TS=("endocrine disorder" OR "diabetes mellitus" OR "thyroid disease" OR "adrenal disorder" OR "pituitary disorder" OR "growth disorder" OR "metabolic syndrome" OR obesity) AND TS=(pediatric OR child OR adolescen OR infant OR youth OR teen) AND TS=(severity OR "clinical outcome" OR hospitalization OR ICU OR "intensive care" OR mortality OR death OR complication)
Cochrane Library	([mh COVID-19] OR "COVID-19" OR "SARS-CoV-2" OR "Coronavirus disease 2019") AND ([mh Endocrine System Diseases] OR "endocrine disorder" OR "diabetes mellitus" OR "thyroid disease" OR "adrenal disorder" OR "pituitary disorder" OR "growth disorder" OR "metabolic syndrome" OR "obesity") AND ([mh Child] OR pediatric OR child OR adolescen OR infant OR youth OR teen) AND (severity OR "clinical outcome" OR hospitalization OR ICU OR "intensive care" OR mortality OR death OR complication)

Study Selection Process

All identified records were imported into EndNote X21 (Clarivate, Philadelphia, PA) for duplicate removal. Two independent reviewers screened titles and abstracts against the predefined eligibility criteria. Full-text articles of potentially relevant studies were retrieved and assessed for final inclusion. Any discrepancies between reviewers were resolved through discussion or consultation with a third reviewer when consensus could not be reached. The study selection process was documented in a PRISMA flow diagram, detailing the number of records identified, screened, and excluded at each stage, along with reasons for exclusion.

Data Extraction and Management

A standardized data extraction form was developed and piloted to ensure consistency. Two reviewers independently extracted data from each included study, including study characteristics (author, year, country, design), participant demographics (sample size, age range, gender distribution), endocrine disorders assessed, COVID-19 severity outcomes, and key findings. Discrepancies in extracted data were resolved through re-examination of the source articles and consensus discussions. Extracted data were compiled into evidence tables to facilitate synthesis and comparison across studies.

Risk of Bias Assessment

The methodological quality of included studies was evaluated using the Newcastle-Ottawa Scale (NOS) [9] for observational studies, which assesses selection, comparability, and outcome domains. Two reviewers independently conducted the risk of bias assessments, with disagreements resolved through discussion. Results were summarized narratively and presented in tabular format to highlight variations in study quality.

Data Synthesis and Analysis

Due to the heterogeneity in study designs, populations, and outcome measures, a meta-analysis was deemed inappropriate. Instead, a narrative synthesis was performed. Findings were first categorized by endocrine disorder type (e.g., diabetes, growth disorders, and thyroid dysfunction) and COVID-19-related outcomes (e.g., disease severity and management challenges). Within these categories, themes were identified through an inductive approach by thoroughly reading and coding extracted data to identify recurring patterns, key clinical implications, and variations across studies. Commonalities and discrepancies were then analyzed to identify overarching trends and gaps in the literature.

Ethical Considerations

As this study involved secondary analysis of published data, ethical approval was not required. However, all included studies were reviewed to ensure they reported ethical approvals and participant consents where applicable.

Results

Search Results

**Figure 1:**
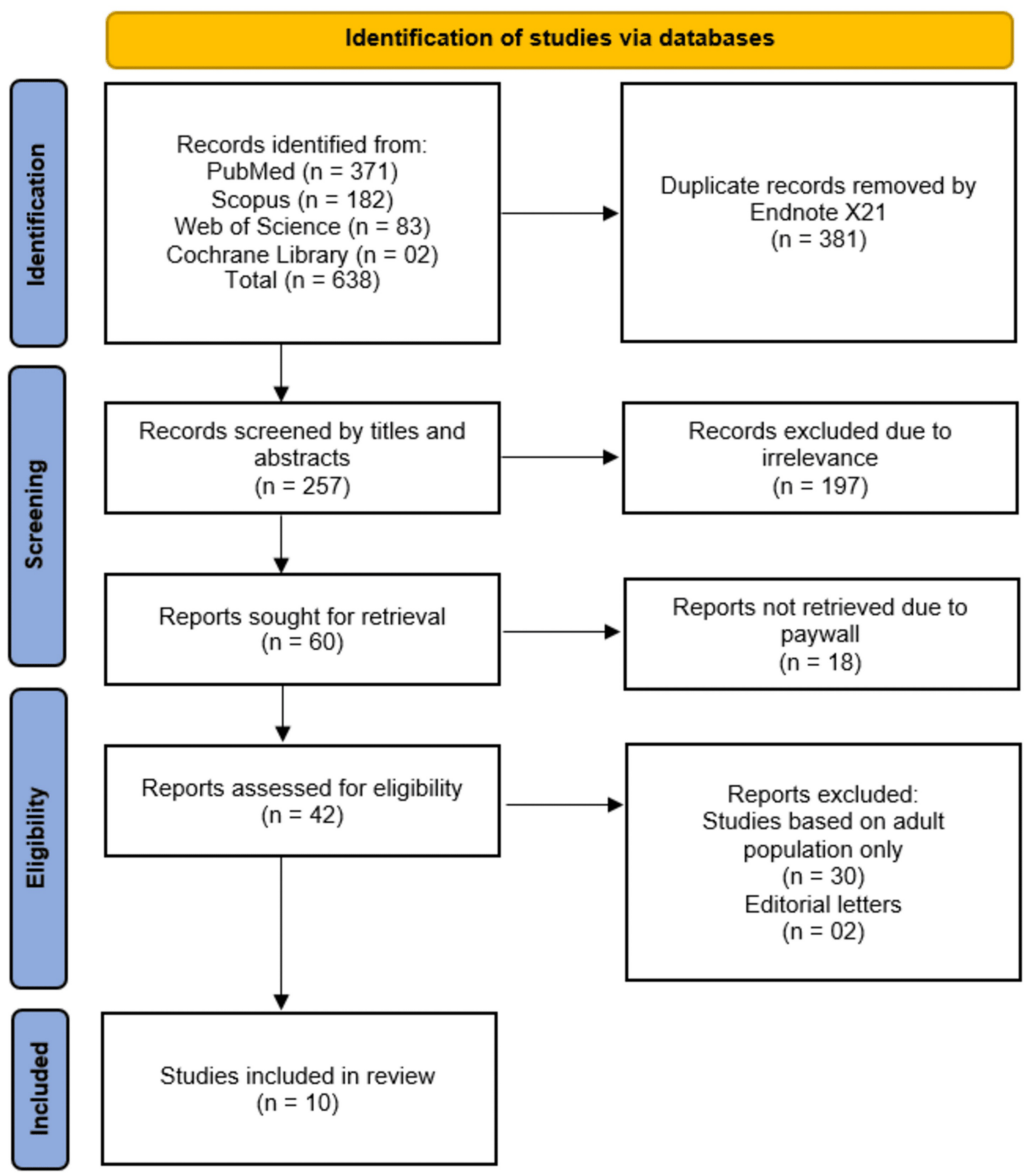
Illustration of studies selection process using the PRISMA flowchart. PRISMA: Preferred Reporting Items for Systematic Reviews and Meta-Analyses.

The systematic search across PubMed (n = 371), Scopus (n = 182), Web of Science (n = 83), and Cochrane Library (n = 2) initially identified 638 records, with 381 duplicates removed. After screening 257 records by title and abstract, 197 were excluded due to irrelevance. Of the remaining 60 full-text reports sought for retrieval, 18 were unavailable, leaving 42 reports assessed for eligibility. Thirty studies were excluded for focusing solely on adult populations, and two were editorial letters, resulting in 10 studies [10-19] meeting inclusion criteria for this review (Figure 1).

Characteristics of Included Studies

The systematic review included 10 studies [10-19] examining the relationship between endocrine disorders and COVID-19 severity in pediatric populations, revealing diverse findings across different endocrine conditions and geographic regions (Table 3). Peinkhofer et al. [10] observed a 35% reduction in growth hormone deficiency (GHD) tests and diagnoses during the pandemic, alongside a 38% increase in central precocious puberty (CPP) cases, predominantly in girls, which the authors attributed to increased screen time, isolation, and anxiety during lockdowns. Similarly, Zachurzok et al. [14] reported increased BMI z-scores in children with endocrine disorders, particularly obesity, during lockdowns, alongside reduced physical activity and increased screen time, though eating habits remained stable. Han et al. [16] corroborated these findings, noting a rise in BMI z-scores during the pandemic compared to pre-pandemic trends, suggesting that activity restrictions significantly impacted growth patterns. These studies collectively highlight the pandemic's indirect effects on endocrine health, particularly through lifestyle disruptions.

**Table 3:** Key characteristics of findings of studies included in this review. CPP: central precocious puberty; PICU: pediatric intensive care unit; FT3: free triiodothyronine; TF: thyroid function.

First author (year)	Country	Study design	Sample size (n)	Age range (years)	Gender distribution	Endocrine disorder(s) studied	COVID-19 severity outcome(s)	Key findings
Peinkhofer et al. (2022) [10]	Italy	Retrospective study	2019: 206 patients (278 tests); 2020: 190 patients (251 tests)	Not specified	Mainly females for CPP increase	Growth hormone deficiency (GHD), central precocious puberty (CPP)	The study focused on endocrine test utilization trends during COVID-19, not severity	35% reduction in GHD tests and 30% reduction in GHD diagnosis during COVID-19; 38% increase in CPP diagnosis, mainly in girls; possible association with increased screen time, isolation, and anxiety during lockdowns
Shafiee et al. (2022) [11]	Multiple countries	Systematic review and meta-analysis including randomized controlled trials, non-randomized trials, and observational studies	7,690,415 (total across 11 studies)	Not specified	Not specified	Type 1 and type 2 diabetes	All-cause mortality, ICU admission, and hospitalization	Type 1 diabetes patients may have a better mortality prognosis; no significant differences in ICU admission or hospitalization between type 1 and type 2 diabetes; findings are heterogeneous and limited by scarce data
Alsulaimani et al. (2022) [12]	Saudi Arabia	Cross-sectional study (survey-based via interviews)	55	Mean age = 12.9 ± 5.8 years (exact range not reported)	Not reported	Congenital adrenal hyperplasia (CAH)	Medication adherence (proxy indicator of management impact during the pandemic)	The COVID-19 pandemic did not significantly affect medication adherence in children with CAH; adherence decreased slightly from 93% to 89% (p = 0.516), with access and prescription challenges increasing after the pandemic onset
Tenenbaum et al. (2021) [13]	Israel	Retrospective, single-center observational study	107	3–18	56.1% male	General pediatric endocrine follow-up (not specific endocrine disorders; focus on growth assessment during the COVID-19 lockdown)	The study focused on the accuracy of home vs. clinic anthropometric measurements during the COVID-19 lockdown	Parents’ home height measurements were accurate except in overweight/obese children; home weight measurements tended to be lower than clinic measurements. With proper guidance, home measurements are suitable for clinical decision-making
Zachurzok et al. (2021) [14]	Poland (Southern Poland: Rzeszów, Kraków, Katowice)	Observational (questionnaire-based with anthropometric measurements before and during the pandemic)	177 (96 females, 81 males)	Mean age = 12.8 ± 2.6 years	96 females, 81 males	Simple obesity, type 1 diabetes mellitus, somatotropin pituitary deficiency on growth hormone therapy, other endocrine/metabolic disorders	Increase in BMI z-scores during lockdown	BMI z-scores increased overall, especially in obese children; decreased physical activity and increased screen time; sleep duration increased; eating habits did not change significantly
Calcaterra et al. (2022) [15]	Italy (Milan)	Retrospective observational study	26	Median = 10.7 (IQR = 5.8–13.3)	19 M/7 F	Thyroid function abnormalities (non-thyroidal illness syndrome, NTIS)	Disease severity score combining the severity scores for each organ involved	88.4% had NTIS; low FT3 common (65.3%); TF related to disease severity, lipemic profile, and insulin resistance; TF may aid in prognosis and long-term management
Han et al. (2021) [16]	Korea	Observational longitudinal study	169	Not specified	Not specified	General endocrine clinic population (not specific to one endocrine disorder)	Change in BMI z-score (proxy for impact of COVID-19 restrictions)	BMI z-score increased during the COVID-19 pandemic (spring 2020) compared to pre-pandemic seasonal trends, suggesting pandemic-related activity restriction impacted growth patterns
Kiess et al. (2021) [17]	Covering multiple countries	Scoping review	Not specified	Children and adolescents	Not specified	Diabetes, obesity, and general endocrine disorders	Severity of the COVID-19 infection; diabetic ketoacidosis frequency	Increased rates of obesity and diabetes post-COVID-19 infection; more severe COVID-19 course in those with obesity and diabetes; well-managed endocrine disorders not linked with higher COVID-19 severity; importance of medication adherence, sick day rules, and telehealth
Banull et al. (2022) [18]	USA (St. Louis Children’s Hospital)	Cross-sectional chart review	390	<25 years (mean: ~10.3 years)	Not reported	Diabetes mellitus (type 1 and type 2), obesity, hypothyroidism, and adrenal insufficiency	Hospital admission, PICU admission	Pre-existing obesity, hypothyroidism, and diabetes mellitus were associated with increased risk of hospital and ICU admission, independent of socioeconomic status
Elbarbary et al. (2021) [19]	51 countries (global survey)	Cross-sectional electronic survey	131 pediatric endocrine centers	Not reported	Not reported	Diabetes, other pediatric endocrine disorders	ICU treatment required for diabetes patients in 21.2% of centers; increased diabetic ketoacidosis in newly diagnosed (44%) and established cases (30%); biopsychosocial concerns, including attempted suicide	Diabetes management was more challenging than other endocrine disorders during COVID-19; increased morbidity risk; psychological distress prevalent; over 20% clinics faced medication/supply shortages; emphasized the need for close healthcare contact and readily available medical supplies

In contrast, studies focusing on diabetes outcomes presented mixed results. Shafiee et al. [11] conducted a meta-analysis of 7,690,415 patients across 11 studies and found that type 1 diabetes patients may have a better mortality prognosis compared to type 2 diabetes patients, though no significant differences were observed in ICU admissions or hospitalizations. However, the authors noted substantial heterogeneity and data limitations. Banull et al. [18] reported that pre-existing obesity, hypothyroidism, and diabetes mellitus were associated with higher risks of hospital and ICU admissions in pediatric COVID-19 patients, independent of socioeconomic status. Elbarbary et al. [19] surveyed 131 pediatric endocrine centers globally and found that diabetes management was particularly challenging during the pandemic, with 21.2% of centers reporting ICU treatment requirements for diabetes patients and increased diabetic ketoacidosis rates in both newly diagnosed (44%) and established cases (30%). These findings underscore the heightened vulnerability of diabetic children to severe COVID-19 outcomes and the pandemic's strain on diabetes care infrastructure.

For other endocrine disorders, the evidence was more varied. Alsulaimani et al. [12] found no significant decline in medication adherence among children with congenital adrenal hyperplasia (CAH), with adherence rates dropping only slightly from 93% to 89% (p = 0.516), though challenges in access and prescriptions were noted. Tenenbaum et al. [13] evaluated the accuracy of home anthropometric measurements during lockdowns and concluded that parental height measurements were reliable for clinical decision-making, except in overweight/obese children, where home weight measurements tended to be underestimated. Calcaterra et al. [15] studied thyroid function abnormalities in pediatric COVID-19 patients and identified non-thyroidal illness syndrome (NTIS) in 88.4% of cases, with low free triiodothyronine (FT3) levels linked to disease severity, lipemic profiles, and insulin resistance, suggesting thyroid function as a potential prognostic marker. Kiess et al. [17] emphasized in their scoping review that while obesity and diabetes were associated with more severe COVID-19 courses, well-managed endocrine disorders did not necessarily increase severity risks, highlighting the importance of medication adherence and telehealth during the pandemic.

Risk of Bias Results

Studies with low risk of bias included Shafiee et al. [11], which demonstrated comprehensive methodology in their meta-analysis, Tenenbaum et al. [13], which employed well-defined anthropometric measurements despite reliance on parental reporting, and Han et al. [16], whose longitudinal design and seasonal adjustments strengthened reliability. Moderate risk of bias was identified in Peinkhofer et al. [10] due to unadjusted confounders in their retrospective cohort; Zachurzok et al. [14] because of self-reported lifestyle data despite objective BMI measures; Calcaterra et al. [15], given their small sample size despite lab-confirmed outcomes; Kiess et al. [17], owing to their narrative synthesis without formal bias assessment; and Banull et al. [18] due to limitations inherent in their retrospective chart review. High risk of bias was noted in Alsulaimani et al. [12] because of their small, non-representative sample and self-reported adherence data, and Elbarbary et al. [19] due to potential non-response bias and lack of confounder adjustment in their global survey (Table 4).

**Table 4:** Risk of bias results using the Newcastle-Ottawa Scale (NOS) tool. SES: socioeconomic status.

Study (author, year)	Selection (Max 4★)	Comparability (Max 2★)	Outcome (Max 3★)	Total stars (Max 9★)	Risk of bias judgment
Peinkhofer et al. (2022) [10]	★★★☆ (3) - Representative sample, but no non-exposed group	★☆ (1) - Limited adjustment for confounders	★★☆ (2) - Clear outcomes, no blinding	6/9	Moderate
Shafiee et al. (2022) [11]	★★★★ (4) - Comprehensive search, duplicate screening	★★ (2) - Adjusted for heterogeneity	★★★ (3) - Rigorous synthesis	9/9	Low
Alsulaimani et al. (2022) [12]	★★☆☆ (2) - Small sample, no representativeness	★☆ (1) - No confounder adjustment	★★☆ (2) - Self-reported adherence	5/9	High
Tenenbaum et al. (2021) [13]	★★★☆ (3) - Single-center but well-defined	★★ (2) - Matched for age/sex	★★☆ (2) - Parental measurements may vary	7/9	Low
Zachurzok et al. (2021) [14]	★★★☆ (3) - Multi-center but self-reported data	★☆ (1) - Partial adjustment	★★☆ (2) - Objective BMI but recall bias	6/9	Moderate
Calcaterra et al. (2022) [15]	★★☆☆ (2) - Small sample, single-center	★★ (2) - Adjusted for severity	★★★ (3) - Lab-confirmed outcomes	7/9	Moderate
Han et al. (2021) [16]	★★★☆ (3) - Longitudinal but limited demographics	★★ (2) - Seasonal adjustments	★★☆ (2) - Reliable BMI trends	7/9	Low
Kiess et al. (2021) [17]	★★★☆ (3) - Broad scope but no protocol	★☆ (1) - Narrative synthesis	★★☆ (2) - No formal bias assessment	6/9	Moderate
Banull et al. (2022) [18]	★★★☆ (3) - Large sample, retrospective	★★ (2) - Adjusted for SES	★★☆ (2) - Chart review limitations	7/9	Moderate
Elbarbary et al. (2021) [19]	★★☆☆ (2) - Global but self-reported	★☆ (1) - No adjustments	★☆☆ (1) - High non-response risk	4/9	High

Discussion

The systematic review examined the interplay between endocrine disorders and COVID-19 severity in pediatric populations, synthesizing evidence from 10 studies across multiple countries. The findings highlight the multifaceted impact of the pandemic on endocrine health, ranging from disruptions in growth patterns and metabolic control to challenges in disease management and healthcare delivery. Below, we discuss these findings in detail, interpret their clinical and public health implications, compare them with existing literature, and outline future research directions.

Impact of COVID-19 on Endocrine Disorders in Children

Growth and pubertal disorders: The pandemic significantly affected growth and pubertal development in children with endocrine disorders. Peinkhofer et al. [10] reported a 35% reduction in GHD diagnoses alongside a 38% increase in CPP cases, particularly in girls. This trend aligns with global reports of accelerated puberty during lockdowns, likely due to increased screen time, reduced physical activity, and psychological stress [20]. Similarly, Zachurzok et al. [14] and Han et al. [16] observed elevated BMI z-scores in children with obesity and other endocrine conditions, reinforcing the role of pandemic-related lifestyle disruptions in metabolic health. These findings are consistent with prior studies linking lockdowns to sedentary behaviors and weight gain in pediatric populations [21].

The reliability of home-based growth monitoring, as assessed by Tenenbaum et al. [13], offers a silver lining: parental height measurements were largely accurate, suggesting that telehealth can support endocrine care when in-person visits are limited. However, the tendency to underestimate weight in overweight children underscores the need for clinician-guided home assessments to avoid misdiagnosis.

Diabetes and metabolic outcomes: The review revealed mixed outcomes for pediatric diabetes during COVID-19. Shafiee et al. [11] found that type 1 diabetes (T1D) patients had better mortality outcomes than type 2 diabetes (T2D) patients, though ICU admission rates were similar. This contrasts with early pandemic data suggesting worse outcomes in T1D due to diabetic ketoacidosis (DKA) [22], but aligns with later meta-analyses showing that well-managed T1D does not necessarily increase COVID-19 severity [23].

Banull et al. [18] and Elbarbary et al. [19] highlighted the heightened vulnerability of diabetic children to severe COVID-19, with obesity and hypothyroidism further exacerbating risks. The surge in DKA cases, both in new-onset (44%) and established (30%) diabetes, reported by Elbarbary et al. [19] mirrors global trends of delayed care-seeking during lockdowns [24]. This underscores the importance of maintaining access to diabetes care during public health crises.

Thyroid and adrenal disorders: Calcaterra et al. [15] identified NTIS in 88.4% of pediatric COVID-19 cases, with low FT3 levels correlating with disease severity. This supports existing evidence that thyroid dysfunction may serve as a prognostic marker in severe infections [25]. For CAH, Alsulaimani et al. [12] found stable medication adherence (93% to 89%, p = 0.516), though supply chain disruptions posed challenges. This resilience contrasts with reports of worsened adherence in chronic diseases during the pandemic [26], possibly reflecting the life-sustaining nature of CAH treatments.

Mechanisms Linking Endocrine Disorders and COVID-19 Severity

Biological pathways: The increased severity of COVID-19 in children with obesity and diabetes may stem from chronic inflammation, insulin resistance, and immune dysregulation [27]. The association between NTIS and severe COVID-19 [15] further suggests that endocrine-immune crosstalk modulates disease outcomes. For example, low FT3 in NTIS may impair cellular repair, exacerbating multi-organ involvement [28].

Psychosocial and healthcare system factors: The pandemic disrupted routine endocrine care, exacerbating disparities in disease management. Elbarbary et al. [19] reported medication shortages in 20% of global endocrine clinics, while Kiess et al. [17] emphasized telehealth as a critical but imperfect substitute for in-person care. Mental health also played a role. Peinkhofer et al. [10] and Zachurzok et al. [14] linked stress and isolation to CPP and obesity, respectively, echoing studies on pandemic-related anxiety in children [29].

Comparison with existing literature: Our findings align with broader research on pandemic-related endocrine disruptions but also reveal unique insights. For instance, the rise in CPP contrasts with initial expectations of delayed puberty due to stress [30], suggesting that environmental factors (e.g., screen time) may override hormonal suppression. Similarly, the stability of CAH adherence [12] challenges assumptions about pandemic-induced non-compliance in chronic diseases.

However, discrepancies exist. While Shafiee et al. [11] reported no ICU admission differences between T1D and T2D, smaller studies found higher DKA rates in T1D [31]. These variations may reflect differences in healthcare systems or pandemic waves studied.

Strengths, Limitations, and Future Directions

This systematic review has several notable strengths. First, the inclusion of diverse study designs, ranging from retrospective cohort analyses to cross-sectional surveys and meta-analyses, enabled a comprehensive synthesis of the evidence. Additionally, the geographic diversity of the studies, encompassing data from Europe, North America, Asia, and the Middle East, enhances the generalizability of the findings to different healthcare settings and populations. The rigorous application of the NOS to assess risk of bias further strengthens the validity of the conclusions, ensuring that methodological quality was systematically evaluated across all included studies.

Despite these strengths, certain limitations must be acknowledged. The heterogeneity in reported outcomes, such as BMI z-scores, DKA rates, and medication adherence metrics, makes direct comparisons challenging and underscores the need for standardized measures in future research. Furthermore, the predominance of retrospective studies introduces potential biases, including recall bias and unmeasured confounding variables, which may affect the reliability of the findings. The reliance on self-reported data in some studies, such as those assessing lifestyle changes during lockdowns, also limits the objectivity of the results.

Moving forward, future research should prioritize prospective longitudinal studies to evaluate the long-term endocrine consequences of COVID-19 in pediatric populations, particularly the sustained effects of pandemic-related lifestyle disruptions on growth, puberty, and metabolic health. Interestingly, studies in adults with endocrine disorders, such as diabetes, obesity, and thyroid dysfunction, have demonstrated similar trends of worsened COVID-19 outcomes, suggesting shared pathophysiological mechanisms (e.g., dysregulated immune responses and chronic inflammation) that may also apply to pediatric populations. However, the long-term implications in children may differ due to developmental vulnerabilities. Additionally, interventional studies are needed to assess strategies for mitigating these disruptions in future public health crises, such as structured telehealth protocols, community-based physical activity programs, and mental health support for at-risk children. Lessons from adult interventions, including targeted glycemic control and hormone replacement therapies, could inform pediatric approaches. Addressing these gaps will be critical for developing evidence-based guidelines to safeguard endocrine health across all age groups during and beyond pandemics.

## Conclusions

This review highlights the profound and multifaceted impact of COVID-19 on pediatric endocrine health, demonstrating significant disruptions in growth patterns, metabolic control, and disease management. The pandemic exacerbated existing endocrine disorders, with notable increases in central precocious puberty, obesity-related complications, and diabetic ketoacidosis, while also revealing the resilience of certain conditions like congenital adrenal hyperplasia in maintaining treatment adherence. These findings underscore the complex interplay between biological, behavioral, and healthcare system factors during public health crises. The consistency of these trends across diverse geographic regions reinforces the global nature of these challenges, though variations in outcomes, such as differences in diabetes severity, highlight the influence of local healthcare infrastructures and pandemic responses.

Moving forward, the lessons learned from this pandemic emphasize the urgent need for adaptive and equitable endocrine care strategies tailored to pediatric populations. Strengthening telehealth infrastructure, ensuring consistent medication access, and integrating mental health support into routine endocrine care should be prioritized to mitigate the long-term consequences of disrupted management. Additionally, future research must adopt prospective, longitudinal designs to elucidate the enduring effects of COVID-19 on childhood endocrine health and to evaluate targeted interventions for at-risk groups. By addressing these gaps, clinicians and policymakers can better safeguard vulnerable pediatric populations in future public health emergencies, ensuring that endocrine care remains both responsive and resilient in the face of systemic disruptions.
